# Double whammy: increased severe primary graft dysfunction after prolonged warm ischemia and inadequate oxygen delivery during heart transplant

**DOI:** 10.3389/frtra.2026.1737467

**Published:** 2026-02-24

**Authors:** Chen Chia Wang, Awab Ahmad, Mark Petrovic, Walter Navid, Christian Eidson, John Trahanas, Aaron M. Williams, Swaroop Bommareddi, Duc Q. Nguyen, Tarek Absi, Eric Quintana, Stephen DeVries, Joey A. Lepore, Matt Warhoover, Aniket S. Rali, Kelly H. Schlendorf, Matthew Bacchetta, Ashish S. Shah, Brian Lima

**Affiliations:** 1 Department of Cardiac Surgery, Vanderbilt University Medical Center, Nashville, TN, United States; 2 Department of Medicine, Vanderbilt University Medical Center, Nashville, TN, United States; 3 Department of Anesthesiology, Vanderbilt University Medical Center, Nashville, TN, United States

**Keywords:** DCD transplant, goal directed perfusion, heart transplant, oxygen delivery, warm ischemia

## Abstract

**Objective:**

This exploratory study examined the relationship between oxygen delivery index (DO2i) during DCD heart transplant (HT), warm ischemic time, and posttransplant outcomes.

**Methods:**

All DCD HT between 10/2021 and 12/2024 using normothermic regional perfusion (*N*RP) at our institution were included. Multiorgan transplants and congenital heart disease patients were excluded. Critical areas—sum of magnitude and duration of DO2i under specific thresholds—were calculated for thresholds 300, 280, and 260 mL/min/m2. Receiver operating characteristics (ROC) analysis dichotomized the critical area into high area (low DO2i) and low area (high DO2i) groups. Patients were then stratified into 4 groups based on high/low functional warm ischemic time (FWIT), and high/low DO2i. Outcomes were compared across groups.

**Results:**

The critical area under 260 mL/min/m2 was the best predictor of severe primary graft dysfunction (PGD). 102 patients met inclusion criteria, and were stratified into four groups based on FWIT above/below 23 min and critical area below/above 1,424 mL/m2 (identified by ROC analysis). 39 (38.2%) patients had low FWIT/ high DO2i, 18 (17.6%) had low FWIT/ low DO2i, 24 (23.5%) had high FWIT/high DO2i, and 21 (20.6%) had high FWIT/low DO2i. Rates of severe PGD were greater in the high FWIT/low DO2i group compared to the low FWIT/high DO2i group (23.8% vs. 0%, *p* = 0.004). Rates of 30-day mortality were higher in the high FWIT/low DO2i group compared to the low FWIT/high DO2i group (14.3% vs. 0%, *p* = 0.039).

**Conclusions:**

Higher oxygen delivery during HT was associated with improved short-term outcomes, and may counteract the myocardial damage from warm ischemia during DCD.

## Introduction

Heart transplantation remains the gold standard therapy for end stage heart failure. However, significantly more patients suffer from end stage heart failure than available organs (1). Donation after circulatory death (DCD) heart transplant has been shown to be a safe and viable method of expanding the donor pool (2, 3). However, DCD heart recovery exposes the allograft to a unique period of warm ischemia after withdrawal of life support in the potential donor. The degree to which these warm ischemic intervals adversely affect post-transplant outcomes remains unclear, with some studies concluding purporting no deleterious impact of prolonged warm ischemia, while others observed increased early mortality (4–6). This is further complicated by varying definitions of clinically significant ischemic intervals (7). Recently published results from our institution found that prolonged functional warm ischemic time (FWIT), defined as the duration between donor oxygen saturation < 80% and initiation of normothermic regional perfusion (NRP), is associated with severe primary graft dysfunction (PGD) for DCD hearts recovered using thoracoabdominal NRP (4).

Goal directed perfusion (GDP) is a strategy in general cardiac surgery of targeting specific goals for perfusion parameters such as oxygen delivery index (DO2i), and has been shown to decrease complications such as acute kidney injury and reduce lengths of stay post cardiac surgery (8–10). The effects of targeting specific DO2i goals during the implanting process on heart transplant outcomes remain largely unstudied. It is plausible that GDP may offer a modifiable target to help improve post-transplant allograft function.

This study represents a novel exploratory analysis examining how DO2i may mitigate the effects of warm ischemia during DCD heart recovery and subsequent transplantation. We hypothesize that two hits—prolonged FWIT in addition to low DO2i during implant—during DCD heart transplant would be associated with increased rates of severe PGD. Therefore, we also hypothesize that among allografts exposed to prolonged FWIT, optimized DO2i during implant would reverse the damage incurred by hypoxemia during the donation process and lead to reduced need for early mechanical support.

## Methods

### Study population

All adult DCD heart transplant recipients at a single institution from October 2021 to December 2024 were reviewed. Only DCD hearts recovered using TA-NRP and transported using static cold storage were included in this study. Allografts transported using a machine perfusion device, multiorgan transplant recipients, and recipients with congenital etiology of heart failure were excluded. Transplants without any cardiopulmonary bypass (CPB) parameters recorded or with missing donor warm ischemic times were also excluded.

### Data definitions and outcomes

Our institutional NRP protocol includes a standard median sternotomy followed by the insertion of a right atrial cannula and direct aortic cannula. Our modified extracorporeal circuit was then used to initiate NRP. The NRP protocols used in this study have been previously described in the literature (1, 2). Our clinical practice is to use NRP for recovery of DCD cardiac allografts, except in instances where NRP is not permitted.

While various definitions of functional warm ischemic time (FWIT) using systolic blood pressure and oxygen saturations exist, our recently published institutional results determined FWIT defined as oxygen saturation < 80% as the best predictor for outcomes (4). Therefore, in this study FWIT was defined as the duration between donor oxygen saturation < 80% and the initiation of NRP during the DCD heart recovery process. DO2i was captured and extracted from our institutional perfusion system (Quantum Perfusion System, Spectrum Medical). DO2i is dependent on several variables and defined as:

DO2i=CardiacOutput×[(1.34×HbxSaO2)+(0.003×PaO2)]/BSA

Hb was hemoglobin, SaO2 was arterial oxygen saturations, PaO2 was partial pressure of oxygen in arterial blood, and BSA was the patient's body surface area.

**Figure 1:**
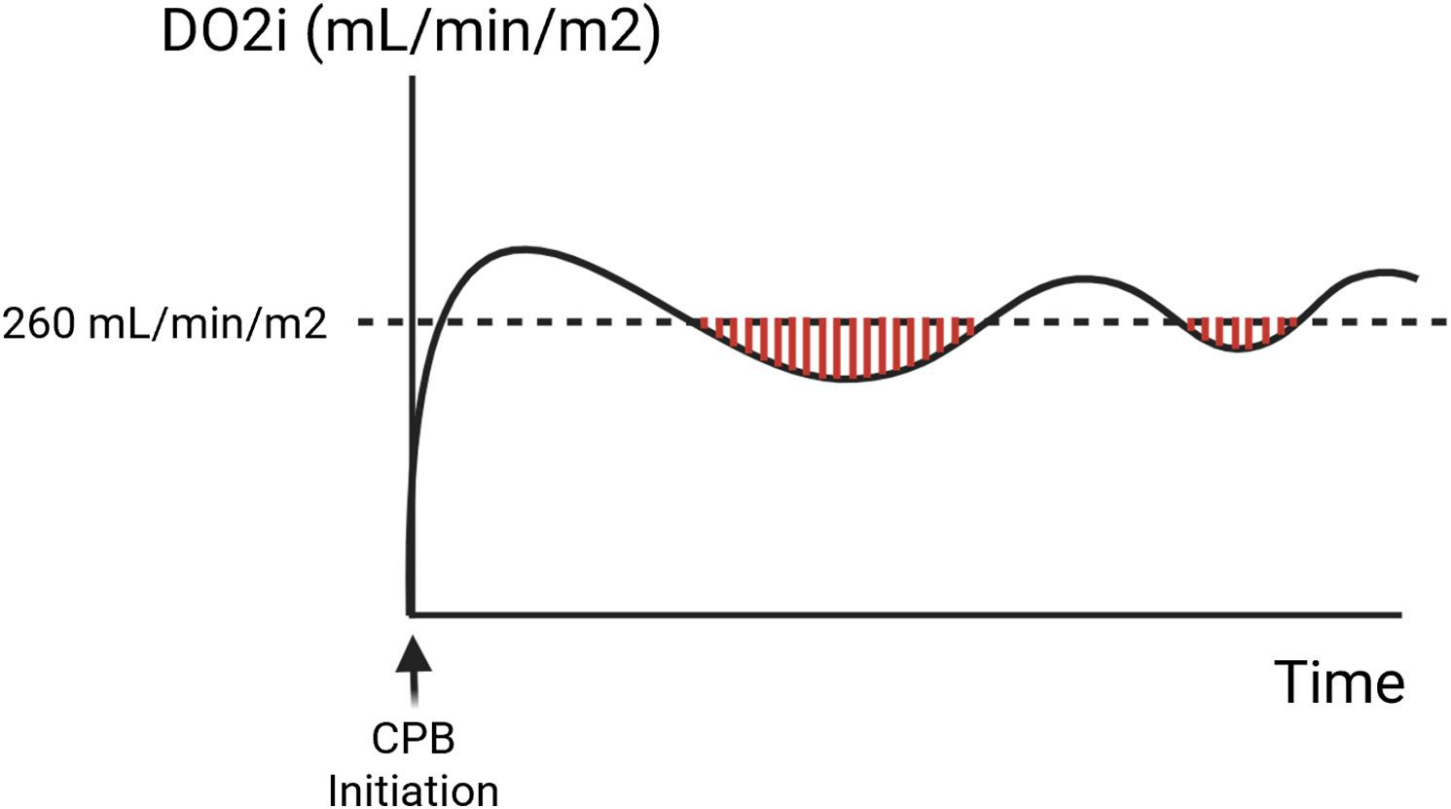
Representative image of critical area of DO2i under a threshold of 260 mL/min/m2. A greater area indicates both greater duration and magnitude below target, thus representing overall lower DO2i.

The critical area—defined as total area under a DO2i threshold and above the continuous perfusion curve—was calculated to represent both magnitude and duration below a specific threshold (Figure 1). This was calculated using the trapezoidal method for integration on a minute by minute basis, where the boundaries were determined by the points where the curve goes above the threshold. In other words, this calculated the area between a continuous curve and a target threshold, but only when the curve is below the threshold. Therefore, a greater critical area for DO2i indicates greater deviation below the perfusion target and/or longer time below the target, which represents lower overall DO2i. Thresholds chosen for DO2i were 300, 280, and 260 mL/min/m2. These thresholds were chosen since the most commonly cited DO2i threshold for GDP in general adult cardiac surgery is 280 mL/min/m2, with some protocols targeting thresholds ranging from 270 to 300 mL/min/m2. (11, 12) Since GDP has not previously been explored in heart transplantation, we selected a higher and lower threshold in addition to the known 280 mL/min/m2 threshold in this exploratory analysis.

Outcomes of interest include primary graft dysfunction (PGD), as defined by the International Society of Heart and Lung Transplantation (ISHLT) consensus statement in 2014 (13), post cardiopulmonary bypass left ventricular ejection fraction (LVEF) and right ventricular function, postoperative cardiac indices, postoperative vasoactive inotrope scores (VIS), acute kidney injury (AKI), LVEF at post operative day 7, duration of mechanical ventilation, ICU and hospital length of stay, and 30-day and 1-year survival. AKI was staged using the KDIGO criteria (14). VIS was calculated as dopamine (µg/kg/min) + dobutamine (µg/kg/min) + 100 * epinephrine (µg/kg/min) + 100 * norepinephrine (µg/kg/min) + 15 * milrinone (µg/kg/min). A common closing follow-up of January 31, 2025, was applied to all patients to ensure complete severe PGD and 30-day mortality data in all patients.

### Statistical analyses

First, univariate logistic regression models were used to compare the strengths of association between DO2i critical areas defined with the three different thresholds and severe PGD. Model performance was compared using the Akaike Information Criterion (AIC), with lower AIC values indicating better model fit relative to model complexity. Once the DO2i critical area definition most predictive of severe PGD was identified, receiver operating characteristic (ROC) curve analysis was used to dichotomize the parameter. Subsequently, patients were stratified into four groups based on the dichotomized DO2i critical area, and 23 min of FWIT. A break point of 23 min for FWIT was selected based on prior literature (4). Demographics and outcomes were compared between the four groups. A multivariate logistic regression model was created to assess the effect of FWIT and DO2i critical area on rates of severe PGD while controlling for known risk factors for worsened outcomes after transplant. The covariates we adjusted for included donor age, total ischemic time, static cold storage modality, recipient ischemic etiology of heart failure, pre-transplant intra-aortic balloon pump support, pre-transplant Impella support, and pre-transplant durable left ventricular assist device support.

A significance level of *p* < 0.05 was used throughout this study. All analysis was conducted using R 4.3.3 (R Core Team). Categorical variables are reported as number (percentage) and compared using Fisher exact test. Continuous variables are reported as mean (standard deviation) and compared using ANOVA. *post-hoc* pairwise comparisons were performed for variables significantly different across four groups, using the Holm-Bonferroni method to adjust for multiple comparisons in categorical variables and Tukey's Honestly Significant Difference test for continuous variables. The Institutional Review Board (IRB) or equivalent ethics committee of the Vanderbilt University Medical Center approved the study protocol and publication of data, under protocol #241909. Patient written consent for the publication of the study data was waived by the IRB due to the retrospective, chart review nature of the study.

## Results

### Oxygen delivery critical area definition and patient stratification

A total of 102 patients met inclusion criteria. The median total warm ischemic time from donor withdrawal to allograft reperfusion on NRP was 26 min (IQR 21–34). The median functional warm ischemic time, defined as oxygen saturation < 80% to reperfusion, was 22 min (IQR 18–29). All other summary statistics of different warm ischemic intervals were shown in Table 1. The average DO2i was 296 mL/min/m2 (IQR 270–319) in the entire cohort. Summary statistics for duration below and critical areas below the pre-specified DO2i thresholds of 260, 280, and 300 mL/min/m2 were also shown in Table 1. On univariate logistic regression, the area under 260 (*p* = 0.018), 280 (*p* = 0.024), and 300 mL/min/m2 (*p* = 0.030) were all significantly associated with severe PGD. The AIC for the model with area under 260 mL/min/m2 was 49.45, compared to an AIC of 50.18 and 50.61 for areas under 280 and 300 mL/min/m2, respectively. This indicates that of the three pre-specified DO2i thresholds, the area under 260 mL/min/m2 was the most predictive of severe PGD. Going forwards, critical area was defined as the DO2i area under 260 mL/min/m2. ROC analysis then found a DO2i area under 260 mL/min/m2 threshold of 1,424 mL/m2 with an area under the curve of 0.82 (95% CI 0.67–0.97), with a sensitivity of 0.88 and specificity of 0.66.

**Table 1:** Median and interquartile range of warm ischemia during DCD allograft recovery and of oxygen delivery index during subsequent transplant in the entire cohort (*N* = 102).

Variable	Statistics
Warm ischemia during DCD recovery	
Total warm ischemia: donor withdrawal to reperfusion, mins	26 (21–34)
Functional warm ischemia, mins	
Oxygen saturations < 80% to reperfusion	22 (18–29)
Systolic blood pressure < 80 mmHg to reperfusion	14 (12–19)
Systolic blood pressure < 50 mmHg to reperfusion	12 (10–15)
Asystolic warm ischemia: declaration of death to reperfusion, mins	9 (8–10)
Oxygen delivery index during transplant	
Average, mL/min/m2	296 (270–319)
Duration below threshold, mins	
260 mL/min/m2	20 (9–47)
280 mL/min/m2	39 (13–79)
300 mL/min/m2	70 (24–113)
Critical area below threshold, mL/m2	
260 mL/min/m2	1,203 (645–2,125)
280 mL/min/m2	1,784 (885–3,428)
300 mL/min/m2	2,980 (1,290–5,330)

The patients were stratified into four groups based on low/high FWIT and critical area below/above 1,424 mL/m2. There were 39 (38.2%) patients with low FWIT/ high DO2i, 18 (17.6%) with low FWIT/ low DO2i, 24 (23.5%) with high FWIT/high DO2i, and 21 (20.6%) with high FWIT/low DO2i.

### Baseline characteristics

Donor age, sex distribution, body mass index (BMI), total ischemic time, and static cold storage technique (ice or 10 °C cooler) were similar between the four groups (Table 2). Recipient age, sex, and BMI were also similar. The proportions of recipients with ischemic cardiomyopathy, re-do sternotomy, and pre-transplant mechanical circulatory support were also comparable. There was also no difference in the distribution of recipient waitlist status at transplant between the groups.

**Table 2:** Donor and recipient characteristics stratified by low/high FWIT during DCD recovery and low/high DO2i during transplant (*N* = 102).

Variable	Low FWIT high DO2i *n* = 39 (38.2%)	Low FWIT low DO2i *n* = 18 (17.6%)	High FWIT high DO2i *n* = 24 (23.5%)	High FWIT low DO2i *n* = 21 (20.6%)	*P* value
Donor					
Sex, female	7 (17.9%)	1 (5.6%)	6 (25.0%)	4 (19.0%)	0.44
Age, y	31.4 (9.3)	28.2 (8.7)	30.4 (9.0)	32.6 (9.7)	0.48
BMI, kg/m^2^	28.2 (9.6)	26.7 (5.0)	29.1 (11.5)	30.8 (12.9)	0.64
Total ischemic time, mins	202 (70)	225 (41)	202 (75)	244 (101)	0.16
Donor distance, nautical miles	318 (203)	324 (283)	299 (272)	385 (324)	0.71
Storage					0.71
Ice	20 (51.3%)	12 (66.7%)	12 (50.0%)	11 (52.4%)	
10 °C cooler	19 (48.7%)	6 (33.3%)	12 (50.0%)	10 (47.6%)	
Recipient					
Sex, female	6 (15.4%)	4 (22.2%)	2 (8.3%)	3 (14.3%)	0.66
Age, y	55.2 (13.4)	54.0 (9.5)	53.4 (15.5)	53.5 (14.0)	0.95
BMI, kg/m^2^	29.2 (4.6)	32.0 (5.0)	28.9 (4.5)	29.2 (5.9)	0.17
Diabetes	16 (41.0%)	9 (50.0%)	8 (33.3%)	12 (57.1%)	0.40
Hypertension	31 (79.5%)	11 (61.1%)	16 (66.7%)	16 (76.2%)	0.44
PHM ratio	0.98 (0.17)	1.00 (0.23)	0.95 (0.19)	1.03 (0.24)	0.66
Etiology, ischemic	13 (33.3%)	9 (50.0%)	3 (12.5%)	6 (28.6%)	0.066
Prior sternotomy	23 (59.0%)	11 (61.1%)	8 (33.3%)	13 (61.9%)	0.15
Pre-transplant MCS	18 (46.2%)	11 (61.1%)	11 (45.8%)	12 (57.1%)	0.66
IABP	4 (10.3%)	2 (11.1%)	2 (8.3%)	1 (4.8%)	0.93
Impella	2 (5.1%)	2 (11.1%)	4 (16.7%)	1 (4.8%)	0.42
LVAD	12 (30.8%)	7 (38.9%)	5 (20.8%)	9 (42.9%)	0.40
ECMO	0 (0%)	0 (0%)	0 (0%)	1 (4.8%)	0.38
Hospitalized	16 (41.0%)	6 (33.3%)	9 (37.5%)	6 (28.6%)	0.74
Waitlist status					0.49
1	2 (5.1%)	0 (0%)	0 (0%)	2 (9.5%)	
2	6 (15.4%)	4 (22.2%)	8 (33.3%)	2 (9.5%)	
3	9 (23.1%)	4 (22.2%)	4 (16.7%)	8 (38.1%)	
4	15 (38.5%)	7 (38.9%)	9 (37.5%)	4 (19.0%)	
6	7 (17.9%)	3 (16.7%)	3 (12.5%)	5 (23.8%)	

Low FWIT: ≤23 min; High FWIT: >23 min; Low DO2i: area under 260 mL/min/m2 > 1,424 mL/m2; High DO2i: area under 260 mL/min/m2 ≤ 1,424 mL/m2. BMI, body mass index; ECMO, extracorporeal membrane oxygenation; IABP, intra-aortic balloon pump; LVAD, left ventricular assist device; MCS, mechanical circulatory support; PHM, predicted heart mass.

### Intraoperative and postoperative outcomes

**Figure 2:**
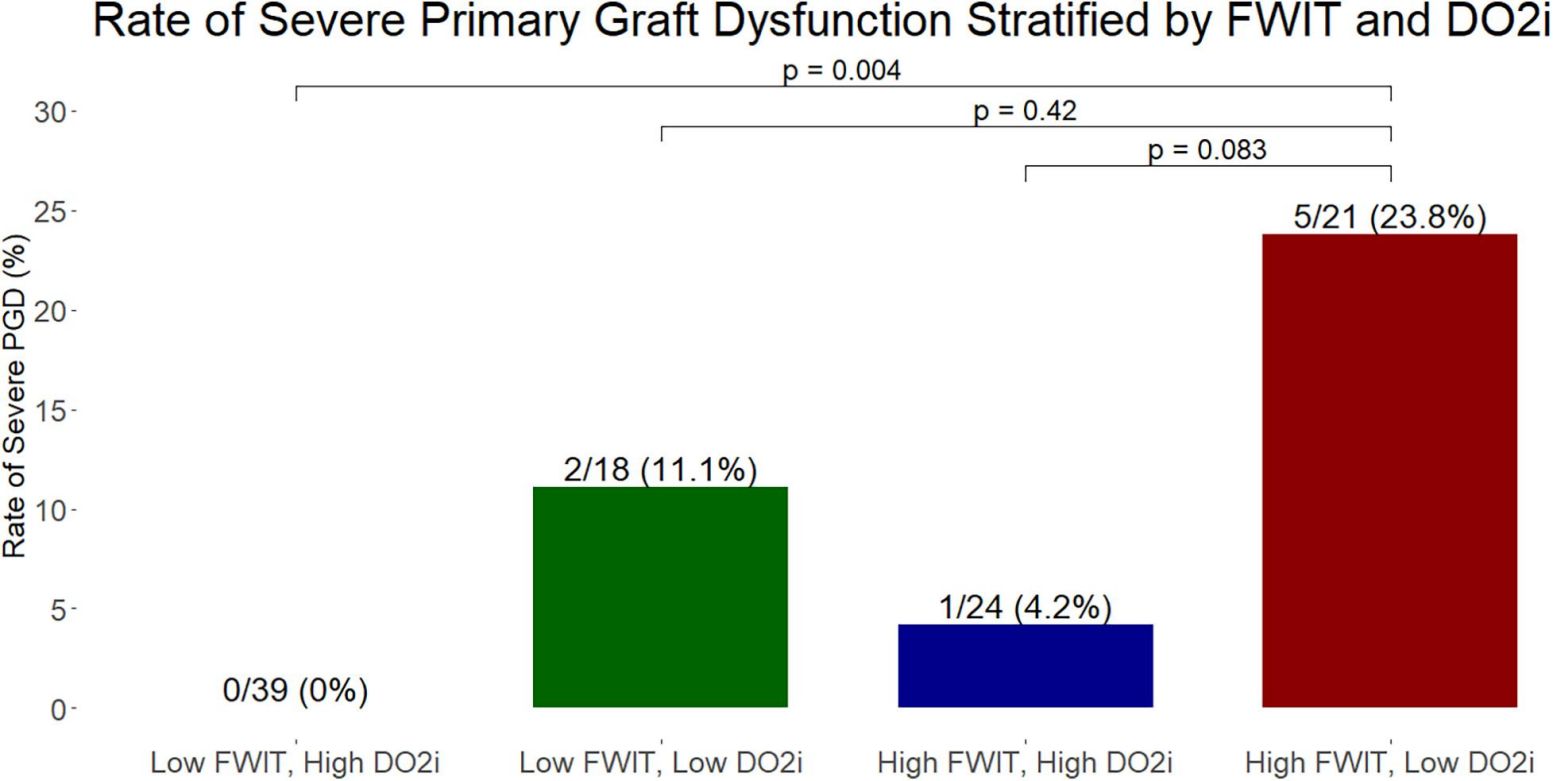
Rates of severe primary graft dysfunction stratified 4 groups: low FWIT and high DO2i (*n* = 39), low FWIT and low DO2i (*n* = 18), high FWIT and high DO2i (*n* = 24), and high FWIT and low DO2i (*n* = 21). The group with both high FWIT and low DO2i had significantly higher rates of severe PGD compared to the group with low FWIT and high DO2i. This data suggests that low DO2i may be the main driver behind severe PGD, and that heart allografts exposed to prolonged FWIT may be successfully recovered with adequate DO2i during implant. Low FWIT: ≤23 min; High FWIT: >23 min; Low DO2i: area under 260 mL/min/m2 > 1,424 mL/m2; High DO2i: area under 260 mL/min/m2 ≤ 1,424 mL/m2. **p < 0.01.

Total bypass length was greater, and minimum hemoglobin and mixed venous saturations were lower, in the two groups with lower DO2i compared to the two groups with high DO2i (Table 3). Pairwise comparisons confirmed that the most drastic differences were between the high FWIT/low DO2i group and both the low FWIT/high DO2i and high FWIT/high DO2i groups. Intraoperative transfusion requirements were also different between the groups, with the high FWIT/low DO2i (average 4,344 mL) requiring significantly higher total transfusions compared to both the low FWIT/high DO2i group (average 2,219 mL) and high FWIT/high DO2i group (average 1,248 mL). When broken down into transfusion requirements of specific blood components, we found that transfusion requirements for packed red blood cells and fresh frozen plasma followed the same pattern as total intraoperative transfusion. Intraoperative LVEF was lower in the high FWIT/low DO2i group compared to the high FWIT/high DO2i group (mean LVEF 49% vs. 55%, *p* = 0.028). Rates of intraoperative severe RV dysfunction were greater in the high FWIT/low DO2i group compared to both the low FWIT/high DO2i (23.8% vs. 5.1%, *p* = 0.038) and high FWIT/high DO2i groups (23.8% vs. 0%, *p* = 0.014). Rates of severe PGD were greater in the high FWIT/low DO2i group compared to the low FWIT/high DO2i group (23.8% vs. 0%, *p* = 0.004) (Figure 2). Rates of 30-day mortality were higher in the high FWIT/low DO2i group compared to the low FWIT/high DO2i group (14.3% vs. 0%, *p* = 0.039). Multivariate regression adjusting for potential confounders demonstrated that increasing FWIT (per minute increase: OR 1.04, 95% CI 1.01–1.09, *p* = 0.013) and lower DO2i captured by increased critical area (per 100 mL/m2 increase: OR 1.05, 95% CI 1.01–1.10, *p* = 0.019) were both independently associated with severe PGD. All *p*-values of pairwise comparisons were shown in Supplementary Table 1.

**Table 3:** Post-transplant outcomes stratified by low/high FWIT during DCD recovery and low/high DO2i during transplant (*N* = 102).

Variable	Low FWIT High DO2i *n* = 39 (38.2%)	Low FWIT Low DO2i *n* = 18 (17.6%)	High FWIT High DO2i *n* = 24 (23.5%)	High FWIT Low DO2i *n* = 21 (20.6%)	*P* value
Outcomes					
Total bypass length	140 (38)	158 (42)	147 (24)	187 (98)	**0**.**016**^a^
Minimum Hb during bypass, g/dL	7.0 (1.5)	6.4 (0.8)	7.1 (1.5)	6.1 (0.8)	**0**.**019**^b^
Minimum SvO2 during bypass, %	58 (12)	51 (7)	59 (12)	48 (16)	**0.003** ^a^ ^,^ ^b^
Intraoperative Total Transfusions, mL	2,219 (1,597)	2,885 (1,350)	1,248 (1,192)	4,344 (4,723)	**<0.001** ^a^ ^,^ ^b^
pRBCs	862 (897)	1,241 (708)	335 (521)	2,083 (2,510)	**<0.001** ^a^ ^,^ ^b^
Platelets	437 (341)	530 (262)	305 (284)	638 (694)	0.058
Fresh frozen plasma	749 (568)	923 (466)	502 (479)	1,326 (1,323)	**0.004** ^a^ ^,^ ^b^
Cryoprecipitate	171 (186)	191 (135)	105 (155)	297 (349)	**0**.**034**^b^
Intraoperative LVEF, %	53 (7)	54 (5)	55 (3)	49 (12)	**0**.**033**^b^
Intraoperative Severe RV Dysfunction	2 (5.1%)	2 (11.1%)	0 (0%)	5 (23.8%)	**0.021** ^a^ ^,^ ^b^
Severe PGD	0 (0%)	2 (11.1%)	1 (4.2%)	5 (23.8%)	**0**.**004**^a^
Ventilator duration, hours	20 (45)	27 (31)	19 (26)	35 (38)	0.43
Cardiac indices, L/min/m2					
ICU arrival	2.9 (0.9)	2.7 (0.8)	2.8 (0.9)	2.9 (0.9)	0.79
24h	3.4 (0.8)	3.2 (1.0)	3.1 (0.8)	3.3 (0.8)	0.71
72h	3.4 (0.8)	3.3 (0.7)	3.2 (0.6)	3.0 (0.9)	0.48
VIS					
ICU arrival	19.4 (8.6)	17.5 (9.1)	16.8 (7.7)	16.1 (6.5)	0.45
24h	11.9 (5.2)	14.3 (8.6)	12.0 (5.6)	13.3 (7.0)	0.55
72h	7.2 (4.4)	8.8 (6.6)	9.4 (7.1)	7.7 (5.9)	0.51
Stage 3 AKI	8 (20.5%)	5 (27.8%)	8 (33.3%)	6 (28.6%)	0.78
Postoperative day 7 LVEF, %	61 (10)	60 (8)	59 (11)	61 (8)	0.87
ICU LOS, days	10 (10)	13 (11)	11 (14)	9 (7)	0.69
Hospital LOS, days	19 (13)	24 (13)	23 (17)	21 (17)	0.66
30-day mortality	0 (0%)	0 (0%)	0 (0%)	3 (14.3%)	**0**.**013**^a^
1-year mortality	1 (2.6%)	1 (5.6%)	3 (12.5%)	3 (14.3%)	0.26

Low FWIT: ≤23 min; High FWIT: >23 min; Low DO2i: area under 260 mL/min/m2 > 1,424 mL/m2; High DO2i: area under 260 mL/min/m2 ≤ 1,424 mL/m2. AKI, acute kidney injury; Hb, hemoglobin; ICU, intensive care unit; LVEF, left ventricular ejection fraction; LOS, length of stay; PGD, primary graft dysfunction; pRBCs, packed red blood cells; SvO2, mixed venous oxygen saturation; VIS, vasoactive inotrope score.

^a^ Indicates significant pairwise difference between the Low FWIT, High DO2i group and High FWIT, Low DO2i group.

^b^ Indicates significant pairwise difference between the High FWIT, High DO2i group and High FWIT, Low DO2i group.

## Discussion

This study served as an exploratory analysis correlating FWIT incurred during the DCD heart recovery process, perfusion parameters during implant, and post-transplant outcomes. The main findings were 1) lower DO2i and prolonged FWIT were independently associated with severe PGD after adjusting for total ischemic time, and 2) optimized DO2i in allografts with prolonged FWIT may mitigate the damage caused by hypoxemia to facilitate organ recovery and reduce the rates of early mechanical support.

These findings partly support our initial hypotheses, where immediate post-transplant graft function seem to differ the most between the group with both high FWIT and low DO2i, compared to the groups with either high FWIT/high DO2i or low FWIT/high DO2i. Specifically, we observed lower, though not statistically significant, rates of severe PGD in allografts with prolonged FWIT when DO2i was high (4.2%) vs. when DO2i was low (23.8%). The significant difference in 30-day mortality, where all cases of mortality were in the high FWIT/low DO2i group, also align with our theory that two hits are required to lead to potentially irreversible allograft damage. While our regression model adjusting for total ischemic time demonstrated that both FWIT and DO2i were independently associated with odds of severe PGD, our four group analysis show that DO2i seems to be a larger driver for severe PGD. When DO2i is high, rates of severe PGD were 0% or 4.2% depending on whether FWIT was low or high, respectively; whereas when DO2i is low, rates of severe PGD were 11.1% or 23.8% depending on whether FWIT was low or high, respectively.

The findings of this study are unprecedented for several reasons. While prolonged FWIT has previously been shown to increase rates of severe PGD (4) and early mortality (6), this study shows that optimizing DO2i during allograft implantation may mitigate the warm ischemic damage. These results also imply that FWIT may not be a significant driver of severe PGD in DCD heart transplants, and that the focus should instead be on optimizing DO2i during the transplant. Therefore, declining DCD allografts based on prolonged warm ischemia may be premature, as those allografts may be salvageable with optimal CPB parameters. Lastly, this study offers clinically applicable, practical takeaways. DO2i can also be altered by upsizing cannulas and adjusting pump flows, and by optimizing hematocrit (transfusions or hemoconcentration) or increasing FiO2. Our data potentially demonstrates that maintaining hemoglobin levels above 7 g/dL throughout the bypass run could help optimize DO2i, as both our groups with low DO2i and minimum hemoglobin levels under 7. However, it was also interesting to note that the high FWIT/low DO2i group had the highest intraoperative transfusions. Given the retrospective nature of the study, we were unable to determine if the transfusions were done in response to low DO2i or for other reasons such as low hemoglobin or prolonged bleeding.

While this was an exploratory analysis, our findings can also spur future investigations and be expanded towards all of heart transplantation. First, we hope this study will spur efforts to unravel the physiological underpinnings behind the effect of DO2i on graft function. We hypothesized that suboptimal perfusion parameters in the transplant recipient could create a “toxic” environment (e.g., high lactate, acidosis) to which the allograft is exposed to after cross clamp removal. Conversely, a healthy environment could potentially facilitate reversal of the ischemic and hypoxemia injury the graft incurred during the donation and transport process. We also believe that oxygen delivery may be a crucial aspect of intraoperative care that can be adjusted to improve outcomes, and be useful for all heart transplants, not limited to DCD. Further investigations into specific perfusion parameter thresholds will be needed to establish perfusion targets, which may vary among different patient populations. Ideally, patient specific perfusion targets can be determined prior to each transplant based on donor/recipient risk factors, and the intraoperative process can move towards a personalized approach.

This study has limitations inherent to a single center, retrospective study. The FWIT threshold was derived from our institutional cohort, and the perioperative management strategies are limited to the standard of care at our institution. Therefore, our findings may not be generalizable to the broad heart transplant population. The small patient population also limited the ability to conduct the robust statistics necessary to determine optimal perfusion thresholds, and we instead based our analyses on arbitrary perfusion targets. However, as an exploratory analysis we believe this still adequately conveys our main message that high oxygen delivery index is associated with improved post-transplant outcomes.

## Conclusions

Goal directed perfusion may be a viable strategy to improve outcomes after DCD NRP heart transplantation. Higher oxygen delivery during heart transplant was associated with decreased rates of severe PGD, and may counteract the potential myocardial damage incurred during warm ischemia of DCD transplant. Future studies are needed to establish and validate perfusion targets based on individual donor/recipient characteristics.

## Data Availability

The raw data supporting the conclusions of this article will be made available by the authors, without undue reservation.
