# Supplementary material for: Double whammy: increased severe primary graft dysfunction after prolonged warm ischemia and inadequate oxygen delivery during heart transplant
Source: Front Transplant. 2026 Feb 24;5:1737467. doi: 10.3389/frtra.2026.1737467 (PMC12971651; doi:10.3389/frtra.2026.1737467)
Supplement: Supplementary file 1 [file Table1.docx]

| **Supplemental Table 1: Post-hoc pairwise comparisons of outcomes with significant differences on four-group analysis (N = 102)** | | | | |
| --- | --- | --- | --- | --- |
| **Total Bypass Length** | | | | |
|  | Low FWIT  High DO2i | Low FWIT  Low DO2i | High FWIT  High DO2i | High FWIT  Low DO2i |
| Low FWIT  High DO2i |  | 0.66 | 0.96 | **0.010** |
| Low FWIT  Low DO2i |  |  | 0.92 | 0.35 |
| High FWIT  High DO2i |  |  |  | 0.071 |
| High FWIT  Low DO2i |  |  |  |  |
| **Minimum Hemoglobin during Bypass** | | | | |
|  | Low FWIT  High DO2i | Low FWIT  Low DO2i | High FWIT  High DO2i | High FWIT  Low DO2i |
| Low FWIT  High DO2i |  | 0.32 | 0.98 | 0.059 |
| Low FWIT  Low DO2i |  |  | 0.23 | 0.94 |
| High FWIT  High DO2i |  |  |  | **0.044** |
| High FWIT  Low DO2i |  |  |  |  |
| **Minimum SvO2 during Bypass** | | | | |
|  | Low FWIT  High DO2i | Low FWIT  Low DO2i | High FWIT  High DO2i | High FWIT  Low DO2i |
| Low FWIT  High DO2i |  | 0.19 | 0.98 | **0.011** |
| Low FWIT  Low DO2i |  |  | 0.13 | 0.85 |
| High FWIT  High DO2i |  |  |  | **0.009** |
| High FWIT  Low DO2i |  |  |  |  |
| **Total Intraoperative Transfusions** | | | | |
|  | Low FWIT  High DO2i | Low FWIT  Low DO2i | High FWIT  High DO2i | High FWIT  Low DO2i |
| Low FWIT  High DO2i |  | 0.78 | 0.44 | **0.011** |
| Low FWIT  Low DO2i |  |  | 0.16 | 0.27 |
| High FWIT  High DO2i |  |  |  | **<0.001** |
| High FWIT  Low DO2i |  |  |  |  |
| **Intraoperative pRBC Transfusions** | | | | |
|  | Low FWIT  High DO2i | Low FWIT  Low DO2i | High FWIT  High DO2i | High FWIT  Low DO2i |
| Low FWIT  High DO2i |  | 0.75 | 0.42 | **0.005** |
| Low FWIT  Low DO2i |  |  | 0.13 | 0.20 |
| High FWIT  High DO2i |  |  |  | **<0.001** |
| High FWIT  Low DO2i |  |  |  |  |
| **Intraoperative Platelet Transfusions** | | | | |
|  | Low FWIT  High DO2i | Low FWIT  Low DO2i | High FWIT  High DO2i | High FWIT  Low DO2i |
| Low FWIT  High DO2i |  | 0.86 | 0.62 | 0.29 |
| Low FWIT  Low DO2i |  |  | 0.32 | 0.85 |
| High FWIT  High DO2i |  |  |  | **0.044** |
| High FWIT  Low DO2i |  |  |  |  |
| **Intraoperative FFP Transfusions** | | | | |
|  | Low FWIT  High DO2i | Low FWIT  Low DO2i | High FWIT  High DO2i | High FWIT  Low DO2i |
| Low FWIT  High DO2i |  | 0.85 | 0.59 | **0.030** |
| Low FWIT  Low DO2i |  |  | 0.29 | 0.35 |
| High FWIT  High DO2i |  |  |  | **0.002** |
| High FWIT  Low DO2i |  |  |  |  |
| **Intraoperative Cryoprecipitate Transfusions** | | | | |
|  | Low FWIT  High DO2i | Low FWIT  Low DO2i | High FWIT  High DO2i | High FWIT  Low DO2i |
| Low FWIT  High DO2i |  | 0.99 | 0.65 | 0.15 |
| Low FWIT  Low DO2i |  |  | 0.58 | 0.43 |
| High FWIT  High DO2i |  |  |  | **0.020** |
| High FWIT  Low DO2i |  |  |  |  |
| **Intraoperative LVEF** | | | | |
|  | Low FWIT  High DO2i | Low FWIT  Low DO2i | High FWIT  High DO2i | High FWIT  Low DO2i |
| Low FWIT  High DO2i |  | >0.99 | 0.87 | 0.085 |
| Low FWIT  Low DO2i |  |  | 0.97 | 0.12 |
| High FWIT  High DO2i |  |  |  | **0.028** |
| High FWIT  Low DO2i |  |  |  |  |
| **Intraoperative Severe RV Dysfunction** | | | | |
|  | Low FWIT  High DO2i | Low FWIT  Low DO2i | High FWIT  High DO2i | High FWIT  Low DO2i |
| Low FWIT  High DO2i |  | 0.58 | 0.52 | **0.038** |
| Low FWIT  Low DO2i |  |  | 0.18 | 0.41 |
| High FWIT  High DO2i |  |  |  | **0.014** |
| High FWIT  Low DO2i |  |  |  |  |
| **Severe PGD** | | | | |
|  | Low FWIT  High DO2i | Low FWIT  Low DO2i | High FWIT  High DO2i | High FWIT  Low DO2i |
| Low FWIT  High DO2i |  | 0.096 | 0.38 | **0.004** |
| Low FWIT  Low DO2i |  |  | 0.57 | 0.42 |
| High FWIT  High DO2i |  |  |  | 0.083 |
| High FWIT  Low DO2i |  |  |  |  |
| **30-Day Mortality** | | | | |
|  | Low FWIT  High DO2i | Low FWIT  Low DO2i | High FWIT  High DO2i | High FWIT  Low DO2i |
| Low FWIT  High DO2i |  | >0.99 | >0.99 | **0.039** |
| Low FWIT  Low DO2i |  |  | >0.99 | 0.23 |
| High FWIT  High DO2i |  |  |  | 0.094 |
| High FWIT  Low DO2i |  |  |  |  |
